# Human colon cancer cells highly express myoferlin to maintain a fit mitochondrial network and escape p53-driven apoptosis

**DOI:** 10.1038/s41389-019-0130-6

**Published:** 2019-03-08

**Authors:** Gilles Rademaker, Brunella Costanza, Justine Bellier, Michael Herfs, Raphaël Peiffer, Ferman Agirman, Naïma Maloujahmoum, Yvette Habraken, Philippe Delvenne, Akeila Bellahcène, Vincent Castronovo, Olivier Peulen

**Affiliations:** 1 Metastasis Research Laboratory, GIGA Cancer, University of Liège, Liège, Belgium; 2 Laboratory of Experimental Pathology, GIGA Cancer, University of Liège, Liège, Belgium; 3 Laboratory of Virology and Immunology, GIGA Molecular Biology of Disease, University of Liège, Liège, Belgium; 4 Pathology Department, Liège University Hospital, Liège, Belgium

**Keywords:** Gastrointestinal cancer, Cell biology

## Abstract

Colon adenocarcinoma is the third most commonly diagnosed cancer and the second deadliest one. Metabolic reprogramming, described as an emerging hallmark of malignant cells, includes the predominant use of glycolysis to produce energy. Recent studies demonstrated that mitochondrial electron transport chain inhibitor reduced colon cancer tumour growth. Accumulating evidence show that myoferlin, a member of the ferlin family, is highly expressed in several cancer types, where it acts as a tumour promoter and participates in the metabolic rewiring towards oxidative metabolism. In this study, we showed that myoferlin expression in colon cancer lesions is associated with low patient survival and is higher than in non-tumoural adjacent tissue. Human colon cancer cells silenced for myoferlin exhibit a reduced oxidative phosphorylation activity associated with mitochondrial fission leading, ROS accumulation, decreased cell growth, and increased apoptosis. We observed the triggering of a DNA damage response culminating to a cell cycle arrest in wild-type p53 cells. The use of a p53 null cell line or a compound able to restore p53 activity (Prima-1) reverted the effects induced by myoferlin silencing, confirming the involvement of p53. The recent identification of a compound interacting with a myoferlin C2 domain and bearing anticancer potency identifies, together with our demonstration, this protein as a suitable new therapeutic target in colon cancer.

## Introduction

Despite an encouraging drop in incidence and mortality, colon cancer is the third most common diagnosed cancer independently of gender^1^. The last GLOBOCAN survey revealed that 10% of new cancer cases are colon cancer^2^. In contrast to what is observed in older people (>50 years), incidence and death rates among younger people continue to rise^1^, making this malignancy the second with the highest mortality rate^2^, and therefore encouraging us to sustain our research efforts.

Cancer cells require catabolites to produce energy and biomass. Metabolic reprogramming of cancer cells includes the predominant use of glycolysis to produce energy (Warburg effect)^3^. However, oxidative phosphorylation (OXPHOS) is also an essential part of their metabolism as it was described to support growth, invasiveness and confer resistance to chemotherapy in several cancer types, including colon^4–6^. Energy metabolism reprogramming, an emerging hallmark of cancer, is necessary for tumour initiation and progression^7^. As such, targeting mitochondrial metabolism appears as a sound potential approach^8^. Accordingly, a recent study demonstrated that a mitochondrial electron transport chain inhibitor (VLX600) reduced colon cancer tumour growth^9^.

Energy metabolism is not only driven by intracellular enzymes but is also conditioned by the intracellular availability of nutrients uptaken through specific transporters. Their abundance at plasma membrane is controlled by several steps, including exocytosis, endocytosis, and recycling. These processes require myoferlin, a 230-kDa multiple C2-domain ferlin family member protein, mainly known for its function in myoblast membrane fusion^10,11^. Previously, we have described the high expression of myoferlin in several cancers^12,13^ and its involvement in cancer cell plasma membrane biology such as endocytosis, membrane receptor recycling, exocytosis, and exosome formation^13–15^. In a metabolic context, we have reported myoferlin as a regulator of lipid metabolism and of mitochondrial dynamics^16,17^. However, its mechanism of action remains poorly understood and unexplored in colon cancer.

In the continuity of our previous studies aiming at showing the importance of myoferlin in cancer aggressiveness and inspired by the lack of information regarding its expression in colon cancer, we have sought to investigate this protein in this context. We have found out that myoferlin expression is highly expressed in colon cancer and correlates with patient survival. We also have showed that myoferlin is required to maintain a high OXPHOS activity and an organised mitochondrial network. We have discovered, for the first time, that myoferlin silencing produces a DNA damage response and a p53-dependent cell cycle arrest.

## Results

### High myoferlin expression in colon cancer lesions is associated with low survival

**Fig. 1:**
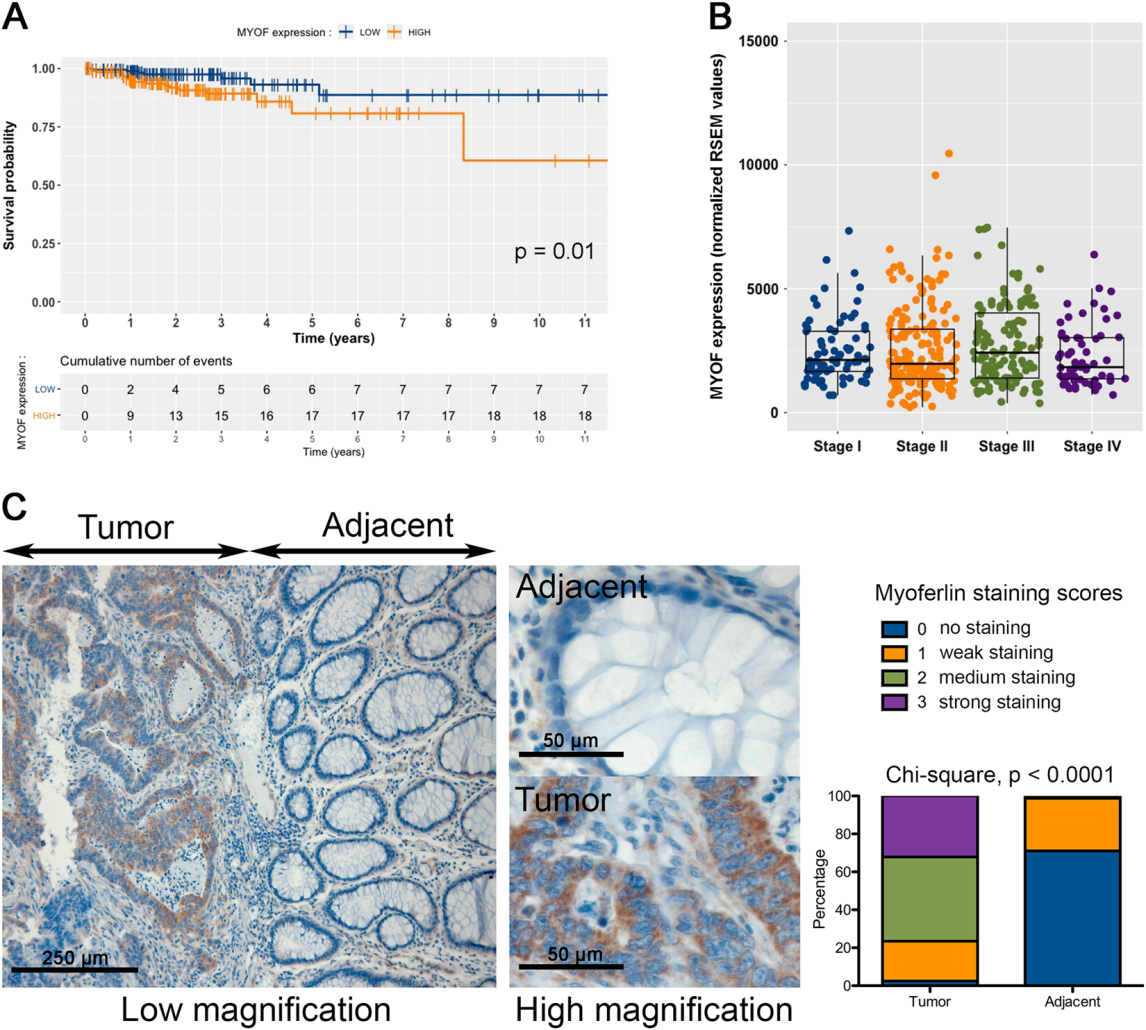
High myoferlin expression is associated with lower survival in colon cancer patients. **a** TCGA-COAD data (http://cancergenome.nih.gov/) were analysed for disease-specific survival according to their myoferlin gene expression split into low (*N* = 220) and high (*N* = 220) according to the median. Kaplan–Meier curves were calculated for each group and a log-rank probability was calculated. **b** Myoferlin gene expression was analysed according to the prognostic stage. **c** Twenty-eight COAD sections were stained and scored for myoferlin (0—no staining, 1—faint staining, 2—medium staining, 3—strong staining). Scores were evaluated for tumour tissue and healthy adjacent

Puzzled by the lack of information regarding myoferlin expression in colon cancer, we decided to mine the PrognoScan databanks^18^ to evaluate the consequence of myoferlin expression on colon cancer patient overall and disease-specific survival. We found a highly significant Cox *P* values (respectively, *P* < 0.01 and *P* < 0.001) in the GSE17536 datasets (*n* = 177). This result was validated using the TCGA-COAD data using the OncoLnc tool^19^. Kaplan–Meier analysis indicated that myoferlin expression in the malignant colon lesions was significantly associated with disease-specific survival. Patients with a high myoferlin expression have a significantly shorter survival time than in patients with low myoferlin expression (*n* = 440, *P* = 0.01—Fig. 1a). The same association was recently shown in breast^16^, kidney^20^, and pancreas^17^ cancers. Using TCGA-COAD data, we evaluated myoferlin gene expression according to prognostic stage (Fig. 1b) and Tumour, Node, Metastasis (TNM) categories (Supplementary figure S1). Neither the prognostic stage nor any of the TNM categories were associated with the myoferlin gene expression. In order to determine whether myoferlin was an independent prognostic factor, we performed a multivariate Cox survival analysis with clinical covariates (Supplementary Table 1). Myoferlin highest percentile was a significant (*P* = 0.039) factor in the prediction of the disease-specific survival with a hazard ratio of 2.618.

We then sought to evaluate myoferlin abundance in a collection of colon cancer specimens (*n* = 28). Myoferlin was strongly stained (mainly scores 2 and 3–76% of the cases) in the cytoplasm of cancer cells, whereas no or faintly detectable (mainly scores 0) in adjacent non-tumoural tissue (Fig. 1c).

### Myoferlin is required to maintain a high OXPHOS activity and an organised mitochondrial network

**Fig. 2:**
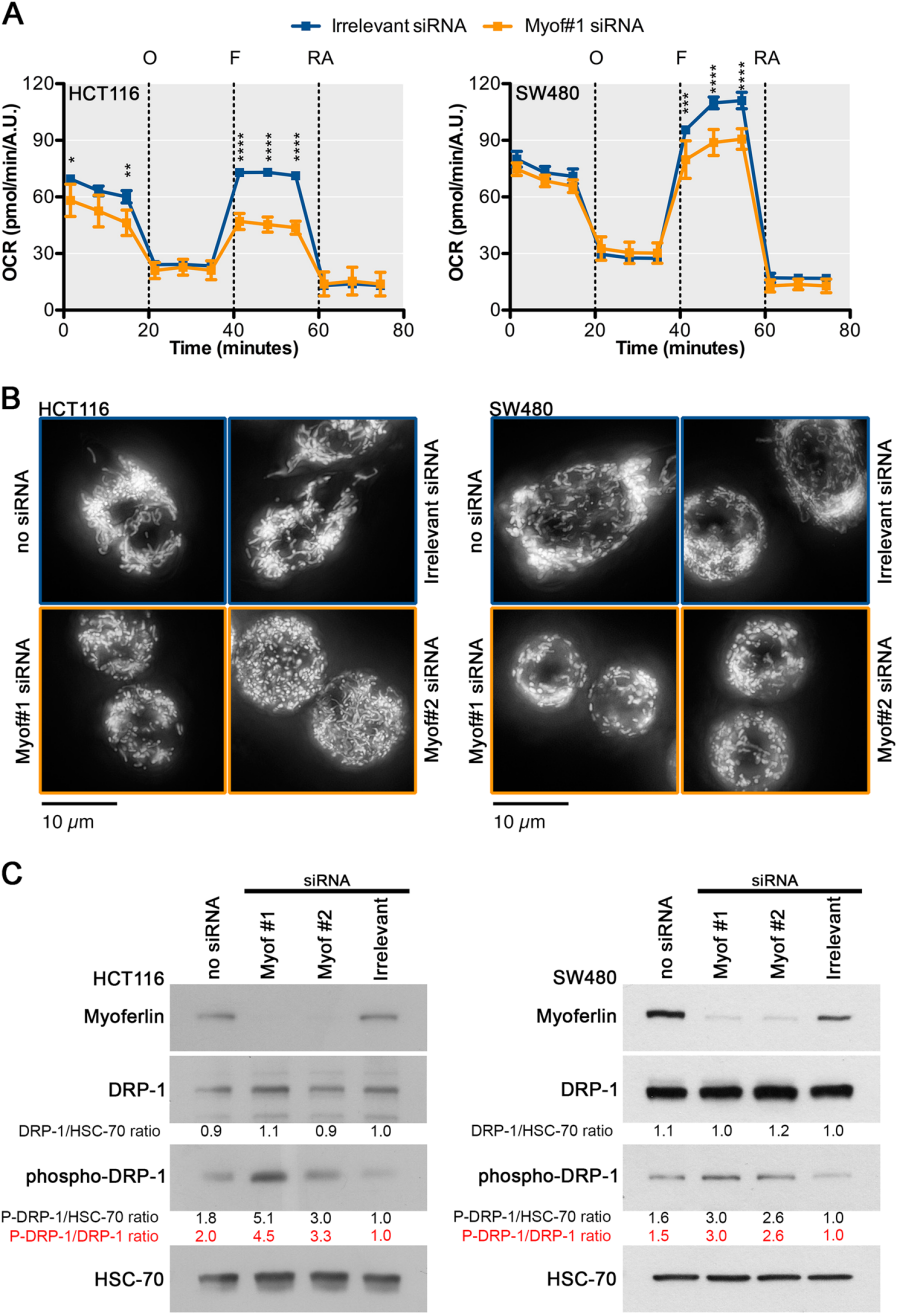
Myoferlin silencing in COAD cell lines reduces oxygen consumption rate (OCR) and disorganises mitochondrial network. **a** Kinetic OCR response of HCT116 and SW480 to oligomycin (O, 1 μM), carbonyl cyanide-*p*-trifluoromethoxyphenylhydrazone (F, 1.0 μM), rotenone, and antimycin A mix (RA, 0.5 μM each). Upon assay completion, cell number was evaluated using Hoechst incorporation (arbitrary unit, A.U.). **b** Tetramethyl rhodamine ethyl ester (TMRE) was used to stain mitochondria in HCT116 and SW480 living cells. At 48 h post-transfection, cells were seeded in μ-Slides 8-well at low confluence, then loaded with TMRE (1 nM). **c** Immunodetection of dynamin-related protein (DRP)-1 or phospho-DRP-1 in myoferlin-silenced HCT116 and SW480 cell lines. Total protein extracts (10 μg) were subjected to sodium dodecyl sulphate–polyacrylamide gel electrophoresis followed by western blot analysis with specific antibodies against myoferlin, DRP-1, or phospho-DRP-1 (ser616). HSC-70 was used as a loading control. One representative experiment out of three is illustrated. Each data point represents mean ± SD, *n* = 3. ****P < 0.0001, ***P < 0.001, **P < 0.01, *P < 0.05

Recently, we have reported that myoferlin contributes to OXPHOS in pancreas cancer^17^. Accordingly, we next evaluated mitochondrial oxygen consumption rate (OCR) and mitochondrial network integrity after myoferlin silencing in colon cancer cell lines expressing highly this protein: HCT116 and SW480 (Supplementary Fig. 2A). We inhibited myoferlin translation using small interfering RNA (siRNA) and monitored the OCR after successive addition of oligomycin, carbonyl cyanide-*p*-trifluoromethoxyphenylhydrazone (FCCP), and rotenone/antimycin A mix. Myoferlin silencing significantly reduced the OCR after FCCP addition in HCT116 and SW480 cell lines (Fig. 2a). Analysis of respiration compartments revealed that maximal OCR was significantly reduced upon myoferlin silencing (Supplementary Figure S2A and S2G). In the HCT116 cell line, this reduction was already apparent in basal condition (Supplementary Figure S2B), while it was not the case in SW480 cell line (Supplementary Figure S2H). In both cell lines, myoferlin silencing led to a decrease of the metabolic potential (Supplementary Figure S2C and S2I). Because of this significant OCR reduction, we decided to evaluate the mitochondrial morphology (Fig. 2b). In HCT116 and SW480, the tetramethyl rhodamine ethyl ester (TMRE)-stained mitochondria appeared as a dense filamentous network. When myoferlin was silenced, the mitochondrial network disappeared and mitochondria turned into individual round-shape structures. Indeed, we observed a significant decrease of the number of networked mitochondria (Supplementary Figure S2D and S2J), of the mean mitochondrial network length (Supplementary Figure S2E and S2K), and of the ratio between the number of networked and individual mitochondria (Supplementary Figure S2F and S2L) when myoferlin was silenced. The mitochondrial network fragmentation induced by myoferlin silencing was concomitant to a dynamin-related protein (DRP)-1 phosphorylation on serine 616 (Fig. 2c). This phosphorylation is known to be implicated in mitochondrial fission^21^. These results suggest that myoferlin is needed to maintain a high and efficient OXPHOS activity in colon cancer cell lines.

### Myoferlin silencing induces reactive oxygen species (ROS) accumulation, reduces cell growth, and induces apoptosis

**Fig. 3:**
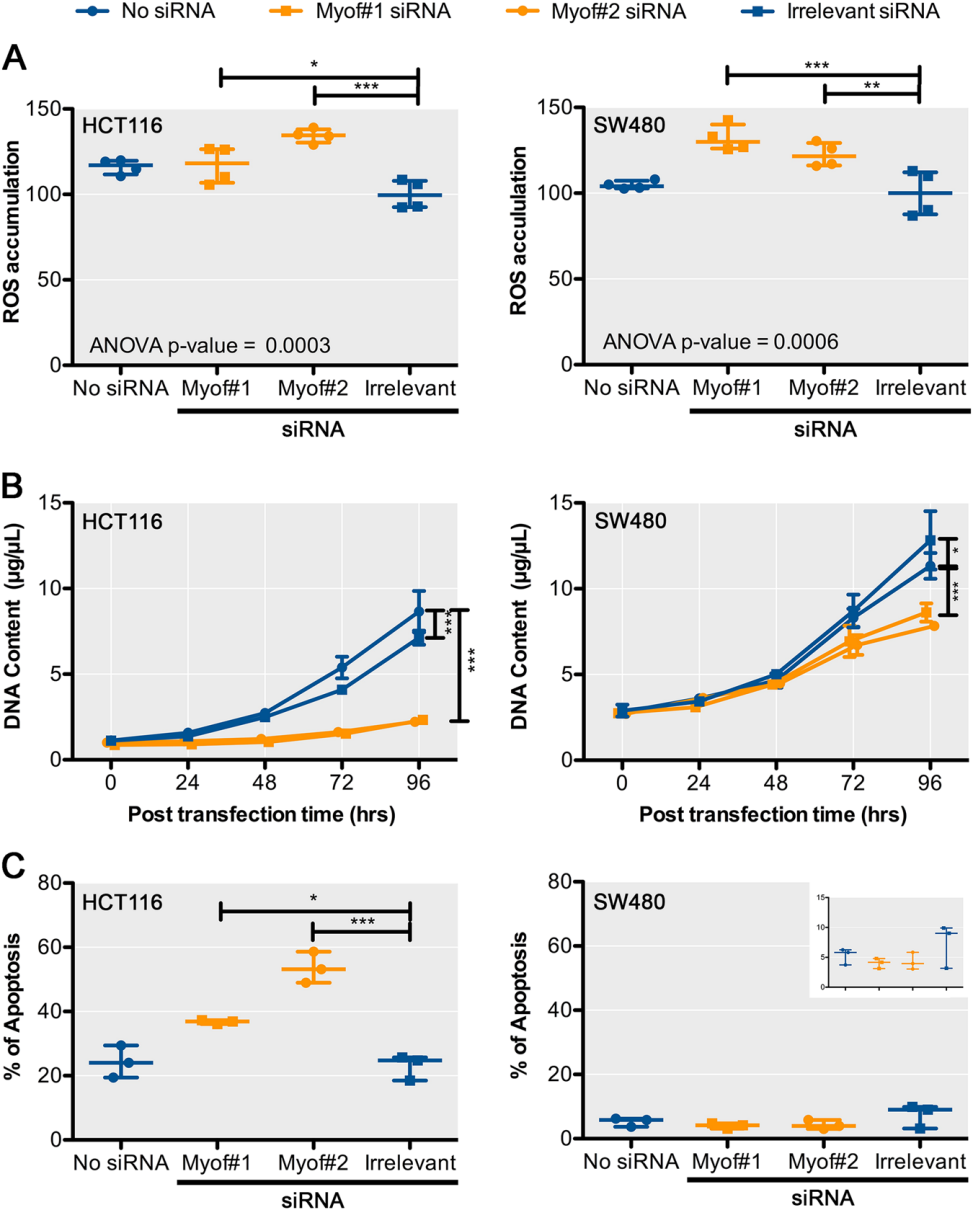
Effects of myoferlin silencing on cell physiology. **a** Reactive oxygen species accumulation in HCT116 and SW480 cells after myoferlin silencing. At 48 h after myoferlin silencing, cells were harvested and loaded with CM-H2DCFDA (2 μM) for 15 min at 37 °C. Then fluorescence was measured by flow cytometry and analysed as median fluorescence intensity. **b** HCT116 and SW480 cell growth was assayed by Hoechst incorporation and indirect DNA quantification. Each data point represents mean ± SD, *n* = 3. **c** Percentage of apoptotic HCT116 or SW480 cells was measured by annexin V/propidium iodide flow cytometry. Insert represents the same data with a specific scale. ***P < 0.001, **P < 0.01, *P < 0.05

We next explored the consequences of the mitochondrial alterations induced by myoferlin silencing in colon cancer cells. First, as mitochondria are the site of electron transport chain and as damaged mitochondria are considered a source of ROS, we measured ROS accumulation. In comparison to irrelevant siRNA, myoferlin silencing produced a 40% increase in mitochondrial ROS accumulation (Fig. 3a and Supplementary Figure S3). The reduced OCR and the accumulation of ROS could influence cell proliferation. Indeed, myoferlin silencing decreased sharply (~75%) the HCT116 cell growth and modestly but significantly (~30%) the SW480 one (Fig. 3b). Accordingly, myoferlin-silenced HCT116 cell produced significantly smaller tumours in a chorioallantoic in ovo model (Supplementary Figure S4). The reduced cell growth of HCT116 cell line was, at least partially, the result of an increased (more than two-fold) apoptosis rate (Fig. 3c). Intriguingly, in SW480 cell line, myoferlin silencing did not alter the percentage of apoptotic cells, remaining low (~5%) (Fig. 3c), indicating another mechanism explaining the low cell growth.

### Myoferlin silencing activates p53 and provokes a cell cycle arrest in HCT116 cell line

**Fig. 4:**
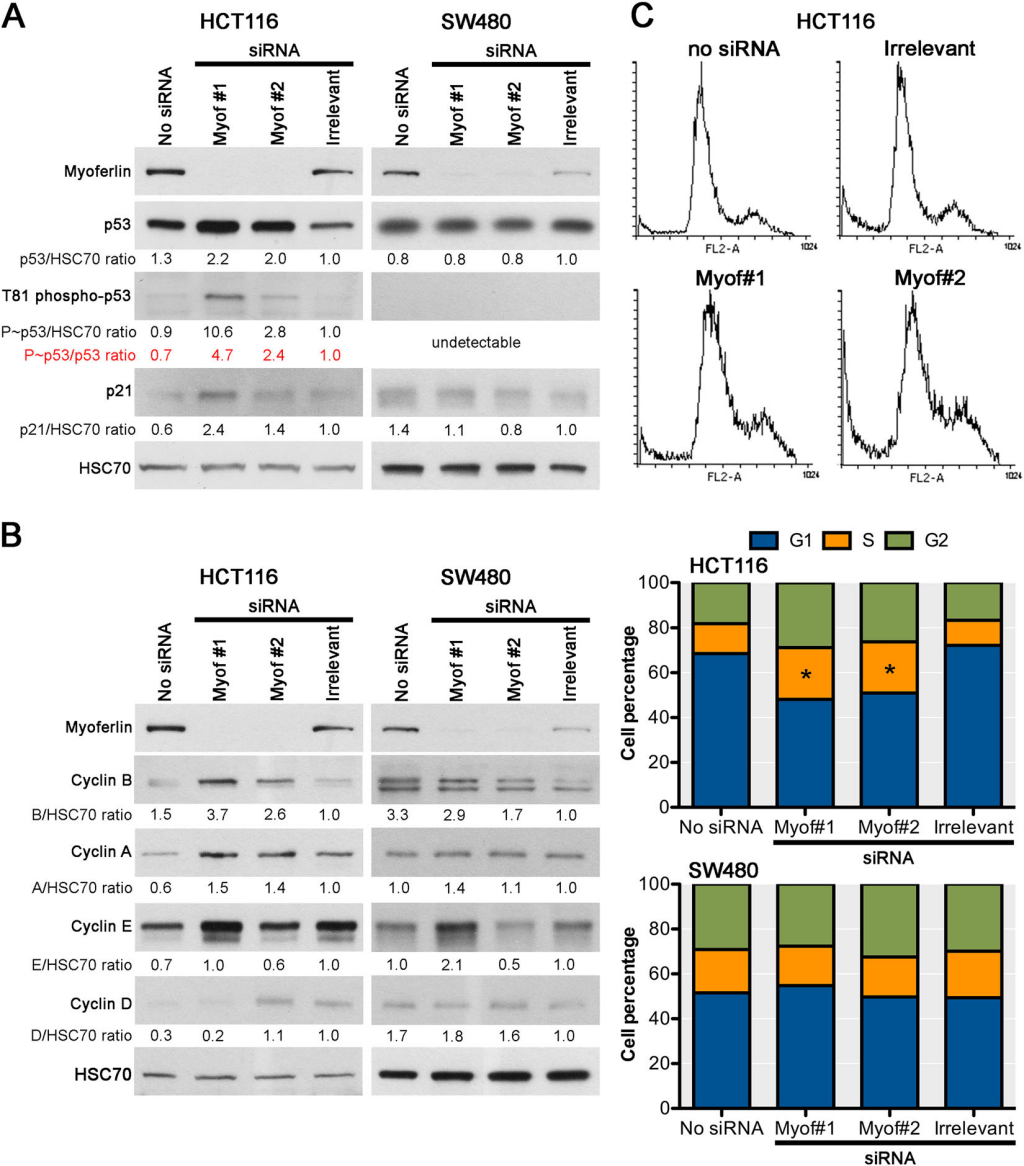
Effects of myoferlin silencing on p53 activation and cell cycle progression. **a** p53 activation by Thr81 phosphorylation and subsequent p21 abundance were evaluated in HCT116 and SW480 48 h after myoferlin silencing. **b** Cyclin abundance was evaluated by western blot in HCT116 and SW480 48 h after myoferlin silencing. Total protein extracts (10 μg) were subjected to sodium dodecyl sulphate–polyacrylamide gel electrophoresis followed by western blot analysis with specific antibodies. HSC-70 was used as a loading control. **c** Cell cycle was analysed by flow cytometry after propidium iodide incorporation in HCT116 and SW480 48 h after myoferlin silencing. Distribution of FL2 fluorescence (propidium iodide) is shown in HCT116. Proportion of cells in G1, S, or G2 is shown in HCT116 and SW480. One representative experiment out of three is illustrated. *P < 0.05

Knowing the opposite p53 status of HCT116 (wild type) and of SW480 (p.R273H; p.P309S), we decided to investigate the role of p53 in the striking behaviour difference between the two cell lines upon myoferlin silencing. We first decided to evaluate the p53 abundance and its threonine 81 phosphorylation status in both cell line after myoferlin silencing (Fig. 4a). Thr81 phosphorylation was evaluated because it is known to be activated by c-Jun N-terminal kinase during ROS-induced DNA damage response^22,23^. In HCT116 cell line (wild-type p53), myoferlin silencing induced a two-fold increase of p53 abundance and a 2.4–4.7-fold increase of its phosphorylation on Thr81, culminating in a 2-fold increase of p21 abundance. Conversely, in SW480 cell line (mutated p53), myoferlin silencing did not alter the p53 abundance. In this cell line, p53 Thr81 phosphorylation was undetectable, as previously described^24^. Based on the p53 activation and p21 abundance increase, suggesting a cell cycle arrest, we decided to analyse the abundance of cyclins (Fig. 4b). In HCT116 cell line, myoferlin silencing provoked an accumulation in cyclin A (S-G2 cyclin) and B (mitotic cyclin). In SW480 cell line, no consistent cyclin modulation was observed after myoferlin depletion. In order to explore the cell cycle, HCT116 and SW480 cell lines were stained with propidium iodide and analysed by flow cytometry (Fig. 4c). It appeared that myoferlin silencing in HCT116 elicited an accumulation of cells in S phase, suggesting a blockage of the S to G2 transition.

### Myoferlin silencing induces a DNA damage response

**Fig. 5:**
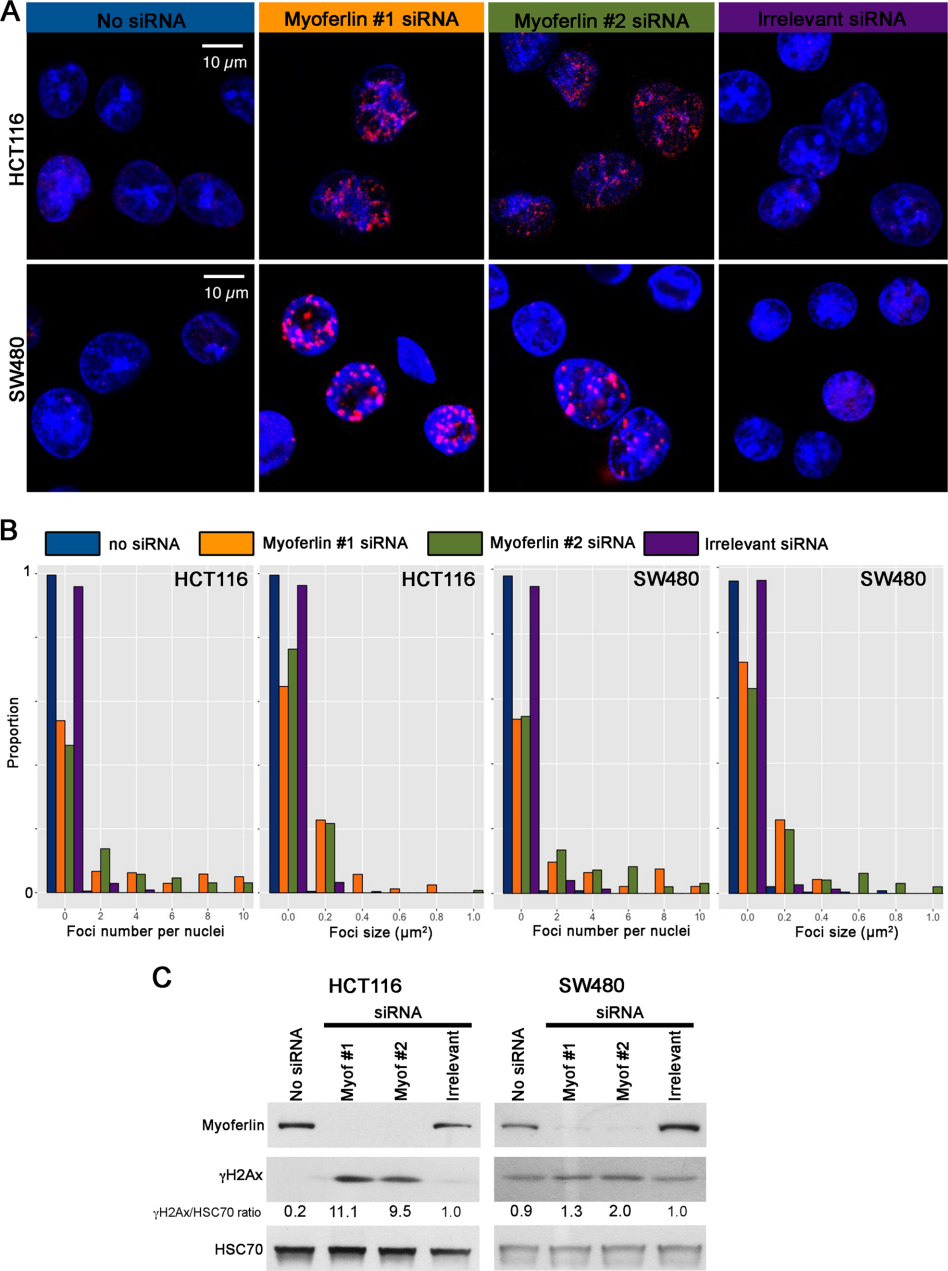
Myoferlin silencing induces a DNA damage response. **a** HCT116 and SW480 cell lines, silenced for myoferlin during 48 h, were stained for γH2Ax and observed under a confocal microscope. **b** γH2Ax foci number and size were quantified using ImageJ. Number and size distributions were established (*n* > 210 nuclei). **c** γH2Ax abundance was evaluated by western blot in HCT116 and SW480 48 h after myoferlin silencing. Total protein extracts (10 μg) were subjected to sodium dodecyl sulphate–polyacrylamide gel electrophoresis followed by western blot analysis with specific antibodies. HSC-70 was used as a loading control

The induced Thr81 phosphorylation of p53 in HCT116 upon myoferlin silencing prompted us to evaluate the cell DNA damage response. HCT116 and SW480 cell lines were immunostained for γH2Ax and observed under a confocal microscope. In both cell lines, γH2Ax foci appeared in the nucleus of myoferlin siRNA-transfected cells (Fig. 5a). Foci quantification indicated an increase of the foci number and of the foci size after myoferlin silencing in both cell lines (Fig. 5b). The accumulation of γH2Ax in transfected cells was also observed by western blot with a ten-fold accumulation in HCT116 and a two-fold accumulation in SW480 (Fig. 5c).

### Myoferlin silencing induces a p53-dependent reduction of cell growth

**Fig. 6:**
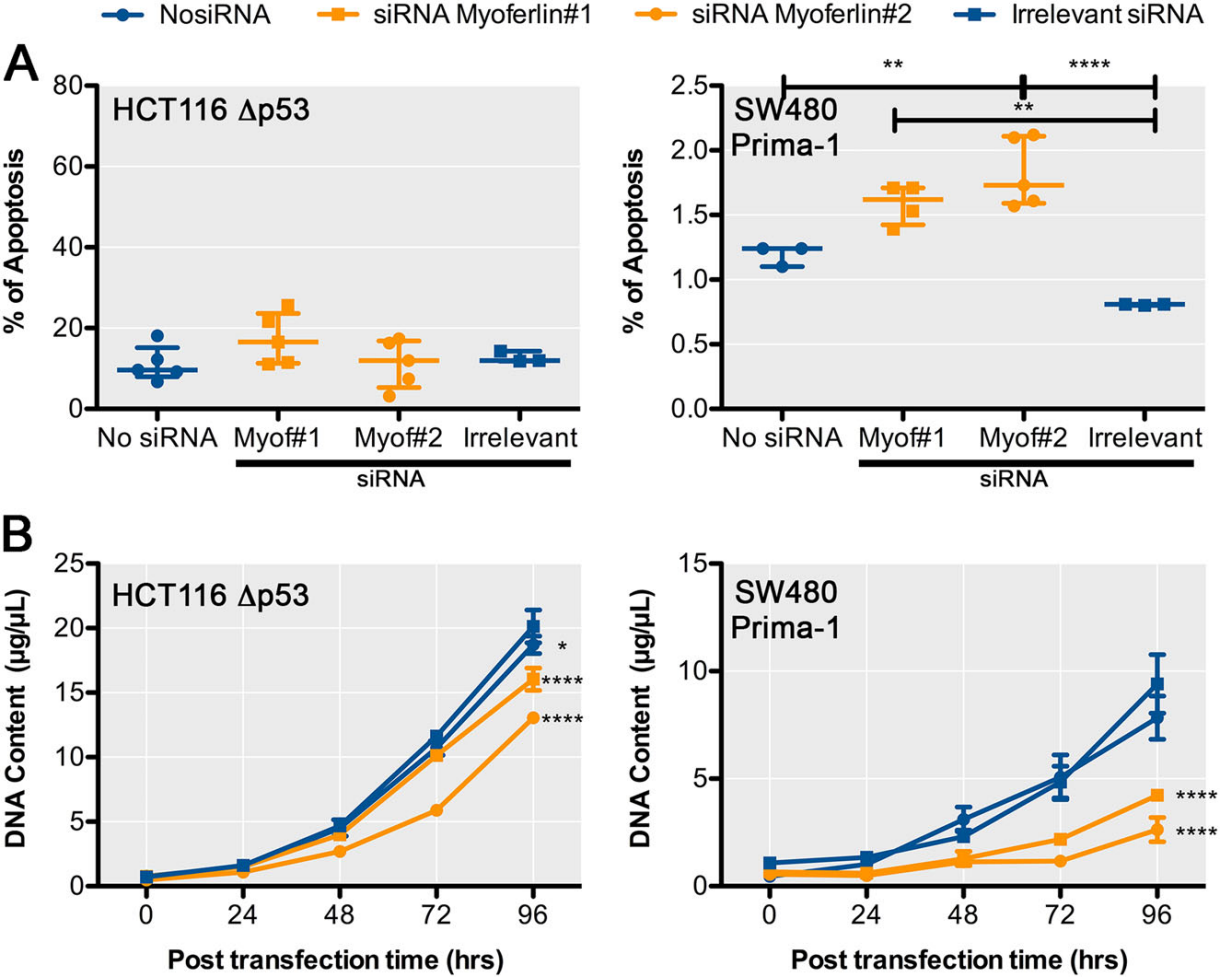
Myoferlin silencing induces reduction of cell growth after p53 restoration. **a** Percentage of apoptotic HCT116 Δp53 cells or SW480 cells treated during 8 h with Prima-1 was measured by annexin V/propidium iodide flow cytometry 48 h after myoferlin silencing. **b** Cell growth of HCT116 Δp53 or SW480 treated during 8 h with Prima-1 was assayed by Hoechst incorporation and indirect DNA quantification 48 h after myoferlin silencing. Each data point represents mean ± SD, *n* = 3. ****P < 0.0001, **P < 0.01, *P < 0.05

**Fig. 7:**
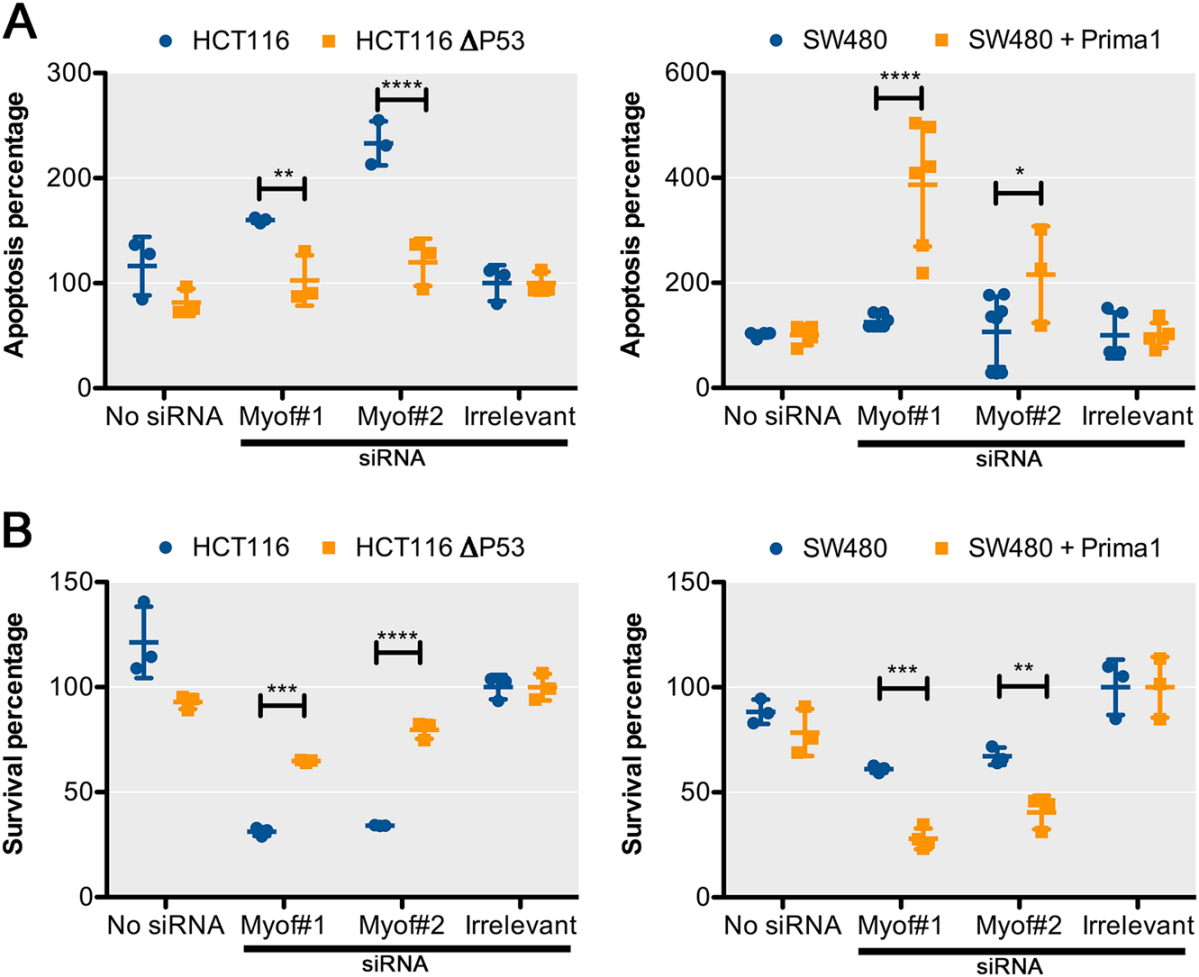
Myoferlin silencing induces a p53-dependent reduction of cell growth. **a** Comparison of apoptosis induction between HCT116 and HCT116 Δp53 cells and between SW480 and Prima-1 treated SW480 48 h after myoferlin silencing. Irrelevant siRNA was used as control (100%). **b** Comparison of 96 h cell growth between HCT116 and HCT116 Δp53 cells and between SW480 and Prima-1 treated SW480 after myoferlin silencing. Irrelevant siRNA was used as control (100%). ****P < 0.0001, ***P < 0.001, **P < 0.01, *P < 0.05

In order to confirm the participation of p53 in the effects of myoferlin silencing, we took advantage of a HCT116 cell line deleted for p53 (HCT116 Δp53) and of SW480 cell treated with a p53-reactivating molecule (Prima-1). HCT116 Δp53 and Prima-1-treated SW480 were characterised for their myoferlin abundance and p53 and p21 status by western blot (Supplementary Figure S5). HCT116 Δp53 seemed to have slightly more myoferlin than the control HCT116 cell line. Myoferlin silencing in HCT116 Δp53 did not alter the percentage of apoptotic cells (Fig. 6a) but partly inhibited the HCT116 Δp53 cell growth (Fig. 6b). In SW480 treated with Prima-1, myoferlin silencing induced a slight but significant increase of the percentage of apoptotic cells (Fig. 6a). In these conditions, cell growth was strongly affected (Fig. 6b). These results clearly showed that p53 deletion from a p53 wild-type cell line (HCT116) or p53 chemical reactivation in a p53 mutant cell line (SW480) completely reversed the apoptotic (Fig. 7a) and growth phenotypes (Fig. 7b) observed after myoferlin silencing. Accordingly, it appeared to us that removing myoferlin should be more advantageous in p53 wild-type patients.

### Myoferlin expression is associated with p53 mutation status in colon cancer patients

**Fig. 8:**
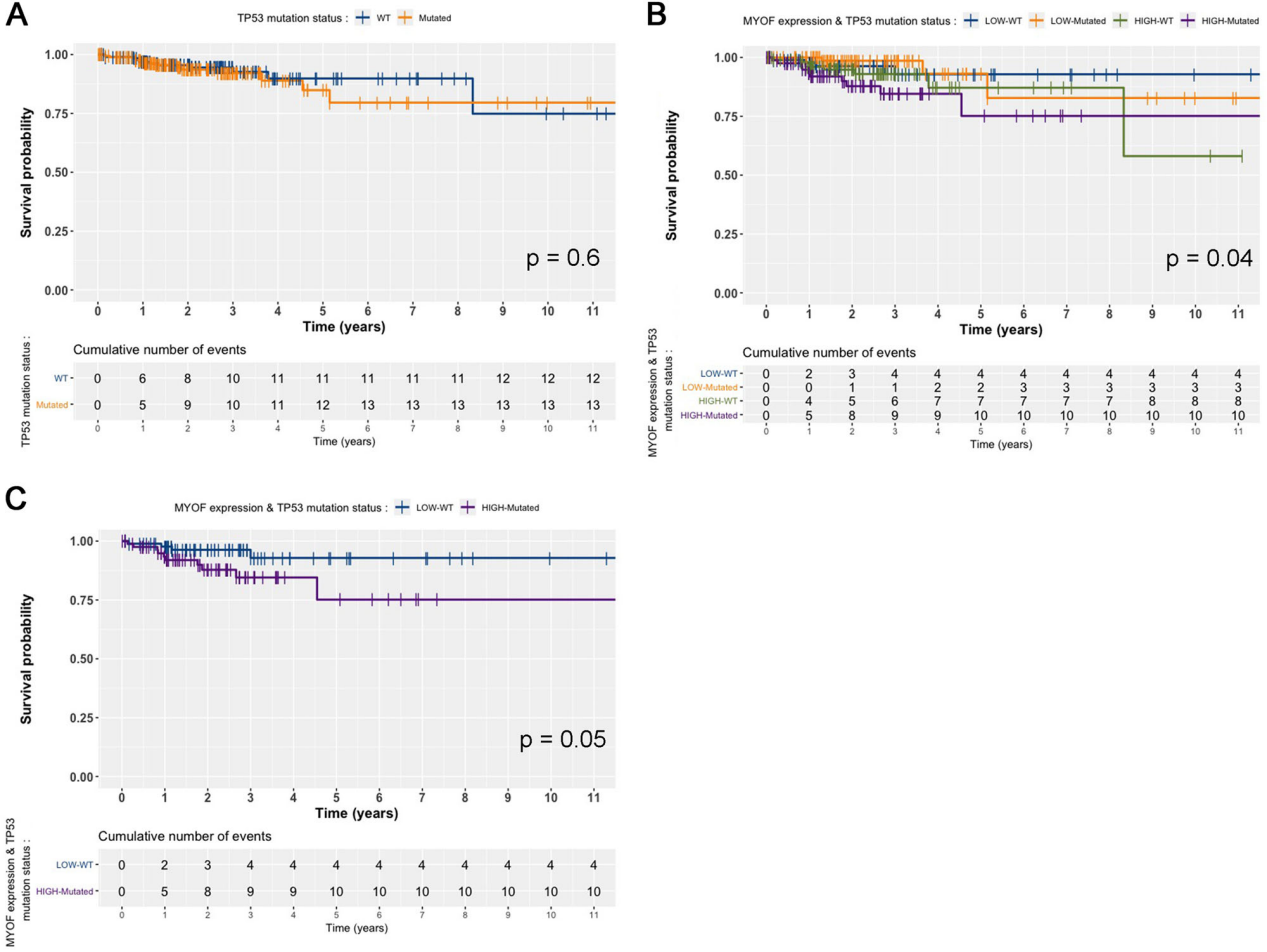
Disease-specific survival is associated with myoferlin expression and p53 mutation status in colon cancer patients. **a** TCGA-COAD data were analysed for survival according to their p53 mutation status. **b**, **c** TCGA-COAD data were analysed for survival according to their p53 mutation status in combination with myoferlin expression. Kaplan–Meier curves were calculated for each group and a log-rank probability was calculated

Inspired by these results, we decided to go back to the TCGA-COAD dataset used initially and mined for an association between myoferlin expression and p53 mutation status. In the present dataset, patient survival was not associated with the p53 mutation status (Fig. 8a). However, when myoferlin expression (low vs high) was combined to the p53 mutation status (wild type vs mutated), a significant difference was found (Fig. 8b). The difference in survival remained statistically significant when myoferlin low–wild-type patients were compared to the myoferlin high–p53-mutated ones (Fig. 8c). Interestingly, myoferlin expression was significantly higher in p53 wild-type patients than in p53-mutated ones (Supplementary Figure S6).

## Discussion

Most cancer cells have a glucose-oriented metabolism, preferring to use glycolysis to OXPHOS to grow. However, a successful growth requests a metabolic flexibility assured by the preservation of several functional catabolic or anaplerotic pathways. The discovery that resistance to treatment rely on OXPHOS in several cancer types^5,25–28^, including colon cancer^5^, recently drew our attention to the importance of identifying the elements rewiring metabolism towards OXPHOS. Targeting OXPHOS in cancer cells appears relevant^8^ as demonstrated by the use of VLX600 in colon cancer cell lines^9^. This compound is an iron chelator^29^ and inhibits respiratory complexes I, II, and IV^9^. Interestingly, due to its antitumour activity in human xenografts^9,30^, this compound was authorised in phase I in patients with refractory advanced solid tumours (clinical trials identifier NCT02222363).

Myoferlin is an emerging tumour-promoting protein described in several cancer types^12,13,20,31–36^, where it was often associated with a poor prognosis^14,16,17,20,33,34,36^. Interestingly, upon interleukin-6 stimulation myoferlin binds to the oncogenic protein signal transducer and activator of transcription factor 3 (STAT3) and participates in its nuclear translocation^37^. In colon cancer, STAT3 activation is associated with an increased proliferation rate, tumour growth, and a reduced survival^38,39^.

We have recently reported that myoferlin contributes to growth factor exocytosis^14^, exosome fusion to recipient cell^15^, lipid metabolism^16^, and mitochondrial fitness^17^. In the current study, we are investigating the involvement of this protein in colon cancer where it was only described at the exosome level^40^. Our results highlight for the first time an association between myoferlin expression and survival of colon cancer patients and show a consistent higher expression of this protein in tumour tissue in comparison with the non-tumoural adjacent one.

Myoferlin silencing in colon cancer cells reduces OXPHOS activity and disturbs the mitochondrial dynamics leading to mitochondrial fission. This process is mainly controlled by DRP-1 phosphorylation catalysed by several kinases, including the mitogen-activated protein kinase extracellular signal–regulated kinase 1/2 (ERK1/2)^41^. Interestingly, myoferlin depletion in hepatocellular carcinoma cells leads to ERK activation^42^ and a cleavable myoferlin is required for ERK phosphorylation in HEK293 cells^43^. We have previously reported this perturbation of the myoferlin-related mitochondrial dynamics in pancreas cancer^17^. However, unlike in pancreas, myoferlin silencing induced a low but consistent and significant accumulation of mitochondrial ROS. The cell-specific growth and the apoptosis observed after myoferlin silencing prompt us to consider their p53 mutation status.

Tumour-suppressor TP53 is the second most frequent mutated gene in colon cancer. TP53 mutations were occasionally described as being associated with low overall survival^44,45^. In our study, TCGA-COAD data did not associate TP53 mutation status to the patient survival confirming previously reported observation^46^. Despite, the lack of consistency in the relation between TP53 mutation and survival, several small molecules were designed for reactivation of p53. These promising compounds are currently tested in clinical trials for hematologic malignancies and suppresses colorectal cancer growth in xenograft mice^47,48^.

P53 protein is the core of a network of pathways in which ROS play critical roles. Upstream of p53, production of ROS is a fundamental mechanism of DNA damage and constitutes a trigger for p53 activation^49^. It appears that the wild-type p53 cells had their cell cycle stalled in S phase upon myoferlin silencing, whereas mutated p53 cells were not influenced. The reversion of the phenotype observed upon myoferlin depletion by modulating p53 activity or presence confirms the role of p53.

To our knowledge, the current study demonstrates for the first time that myoferlin silencing results in DNA damage probably due to ROS production by the fragmented mitochondria^50^. Interestingly, it has been previously shown that therapeutic agents generating ROS are more likely to be toxic for wild-type p53 tumour cells^51^. Accordingly, p53 reactivation increased the efficiency of myoferlin silencing on cell growth inhibition.

The cell growth arrest and the apoptosis generated by myoferlin silencing in p53 efficient cell line could open up new perspectives in the development of multi-modal therapies associating p53 and myoferlin targeting. In light of our results, about 75% of COAD patients exhibit a myoferlin higher expression at the protein level and, in consequence, can be considered as eligible to a myoferlin-targeted therapy. Considering a p53 mutation incidence of about 50%, half of the patients expressing myoferlin could benefit of the proposed multi-modal strategy. Currently, p53 restoration drugs are under clinical trials and C2-domain-targeting compounds are under development^52^. While we were preparing this manuscript, a study reported a small molecule targeting myoferlin with significant antitumour effect on breast cancer and on several other cancer cell types, including pancreas cancer, prostate cancer, and ovarian cancer^53^. This compound exerts promising antimetastatic activity at nanomolar concentration most probably by its interaction with the C2D domain of myoferlin. Together with our results, this discovery adds weight to the potential of myoferlin to be used as a relevant target for new anticancer therapies.

## Materials and methods

### Cells and chemicals

Human colon caner cells HCT116 (ATCC CCL-247) and SW480 cells (ATCC CCL-228) were purchased from ATCC. Cells were authenticated by Short-Tandem Repeat profiling (DSMZ, Braunschweig, Germany). All reagents were purchased from Sigma (Bornem, Belgium) unless mentioned otherwise. Antibodies were purchased from Sigma Life Sciences (Bornem, Belgium): myoferlin (HPA014245); Santa Cruz Biotechnology (Santa Cruz, CA): HSC70 (sc-7298), p21 (sc-6246), DRP-1 (sc-271583), Cyclin B1 (sc-752); Cell Signaling (Danvers, MA): phospho-DRP-1 (Ser616) (4494), phospho-p53 (Thr81) (2676), γH2A.X (9718), Cyclin D1 (2978); Merck-Millipore (Darmstadt, Germany): mitochondria (MAB1273), p53 clone BP53–12 (05–224); BD Biosciences: Cyclin A (611268), Cyclin E (551159).

### Cell culture

HCT116 cells were maintained in Dulbecco’s modified Eagle’s medium supplemented with fetal bovine serum (10% FBS). SW480 cells were cultured in Minimum Essential Medium (MEM) supplemented with FBS (10%), L-glutamine (2 mM), sodium pyruvate (1 mM), and non-essential amino acids for MEM (Gibco #11140-085). Cells were cultured in a 37 °C, 5% CO_2_ incubator. Cells were recently authenticated and used between passage 1 and 10 and checked monthly for mycoplasma.

### SiRNA transfection

HCT116 and SW480 cells were transfected with 40 nM siRNA, respectively, using calcium phosphate or lipofectamine as described previously^17,54^. All experiments were performed 48 h after transfection.

### Western blotting

Cells were lysed in sodium dodecyl sulphate (SDS; 1%) in the presence of protease and phosphatase inhibitors. SDS-polyacrylamide gel electrophoresis were performed as described previously^16^. Band quantifications were performed with the Image Studio Lite software v5.2.5 (LI-COR Biosciences).

### Extracellular flux analysis

All experiments were performed with a Seahorse XFp extracellular flux analyser (Agilent). Cells were seeded (20,000 cells/well) in XFp mini-plates (Agilent) and allowed to attach overnight. Mitochondrial OCR (pmoles/min) were measured as previously described^17^. Results were normalised according the cell number evaluated by Hoechst (2 µg/mL) incorporation. Results shown are representative ones out of three independent experiments.

### Mitochondrial TMRE staining

TMRE was used to stain mitochondria in living cells as described previously^17^. Images were acquired by epifluorescence microscopy as *Z*-stacks with a Nikon A1R microscope equipped with ×100 Oil objective. Mitochondrial network was quantitatively assessed using image analyses with the MiNA Fiji plugin^55^. Results shown are representative ones out of three experiments.

### Immunofluorescence

After siRNA transfection, 5 × 10^4^ cells were seeded on sterile glass coverslips. Forty-eight hours after transfection, cells were washed, fixed, and then blocked as described^17^. Coverslips were incubated overnight at 4 °C with the primary antibody against γH2Ax diluted in phosphate-buffered saline–bovine serum albumin solution. Coverslips were washed and incubated with Alexafluor 546 conjugated secondary antibody for 1 h at room temperature. Sections were mounted following washes and nuclei staining with Hoechst (10 ng/mL). *Z*-stack images were acquired using a Nikon A1R confocal microscope equipped with a Nikon ×100 Oil objective. Results shown are representative ones out of three experiments.

### Mitochondrial ROS measurement

Mitochondrial ROS production was measured by flow cytometry using Mitosox (Invitrogen) fluorescent probe according to the manufacturer’s protocol. Results shown are cumulative ones from three independent experiments.

### Annexin V/propidium iodide staining

Percentage of apoptotic cells was assessed by flow cytometry using fluorescein isothiocyanate-annexin V and propidium iodide staining (BD Biosciences, Franklin Lakes, NJ) according to the manufacturer’s instructions. Results shown are cumulative ones from three independent experiments.

### Cell cycle analysis

Cell were trypsinised, washed once with PBS and then fixed with ice-cold 70% ethanol for 4h at 4 °C. Fixed cells were washed once with PBS, then treated with RNAse (50µg/mL) and stained with propidium iodide (50 µg/mL) for 30 min at room temperature. Cells were analysed by flow cytometry with FACS calibur (BD Biosciences, Franklin Lakes, NJ). Results shown are cumulative ones from three independent experiments.

### Cell growth

Equal number of cells were seeded in complete medium after transfection and harvested after 48 h. The cell numbers were indirectly determined using Hoechst incorporation. Results were expressed as DNA content. Results shown are representative ones out of three experiments.

### Immunochemistry and staining assessment

Primary colon tumours were obtained from our institution Biobank, as formalin-fixed, paraffin-embedded tissue blocks. Sections were stained with antibodies against myoferlin. Sections were then reviewed and scored blindly by three independent investigators (G.R., M.H., and O.P.). Myoferlin scoring was performed by evaluating the intensity (ranging from 0 to 3) of each immunolabelled sample.

### Chorioallantoic membrane assay (CAM)

HCT116 cells were grown on CAM for 7 days as previously mentioned^14,56^ and then tumour volumes have been calculated as an ellipsoid. Ten CAM were grafted in each experimental condition. Results shown are representative ones out of three experiments.

### Statistics

Kaplan–Meier survival curves were established based on TCGA-COAD data. Survival curves were compared using the log-rank test. All other results are reported as means with standard deviation (SD). Two-sided statistical analyses were performed using one-way or two-way analysis of variance depending on the number of grouping factors. Unless mentioned otherwise, group means were compared by unpaired Student’s *t* test or Bonferroni’s post-test according to the group number. Welsch’s correction was applied when homoscedasticity was suspected. *P* < 0.05 was considered as statistically significant. Unlabelled differences between groups were non-significant. All experiments were performed as several independent biological replicates. Statistics were performed using R v3.4^57^.

## Supplementary information

Supplemental legends

Supplemental Figure 1

Supplemental Figure 2

Supplemental Figure 3

Supplemental Figure 4

Supplemental Figure 5

Supplemental Figure 6
