# Correction: Human colon cancer cells highly express myoferlin to maintain a fit mitochondrial network and escape p53-driven apoptosis

**DOI:** 10.1038/s41389-023-00455-5

**Published:** 2023-03-02

**Authors:** Gilles Rademaker, Brunella Costanza, Justine Bellier, Michael Herfs, Raphaël Peiffer, Ferman Agirman, Naïma Maloujahmoum, Yvette Habraken, Philippe Delvenne, Akeila Bellahcène, Vincent Castronovo, Olivier Peulen

**Affiliations:** 1 Metastasis Research Laboratory, GIGA Cancer, University of Liège, Liège, Belgium; 2 Laboratory of Experimental Pathology, GIGA Cancer, University of Liège, Liège, Belgium; 3 Pathology Department, Liège University Hospital, Liège, Belgium; 4 Laboratory of Virology and Immunology, GIGA Molecular Biology of Disease, University of Liège, Liège, Belgium

Correction to: *Oncogenesis* 10.1038/s41389-019-0130-6, published online 08 March 2019

During figure preparation, the same protein samples were used in the western blots depicted in figures 4 and 5. For the sake of completeness, the same myoferlin western blots were included in figures 4 and 5. It has been noted that the western blot duplication can be misleading. Consequently, the duplicated myoferlin western blots have been removed from figures 4 and 5. The corrected figures are presented below.

**Fig. 4:**
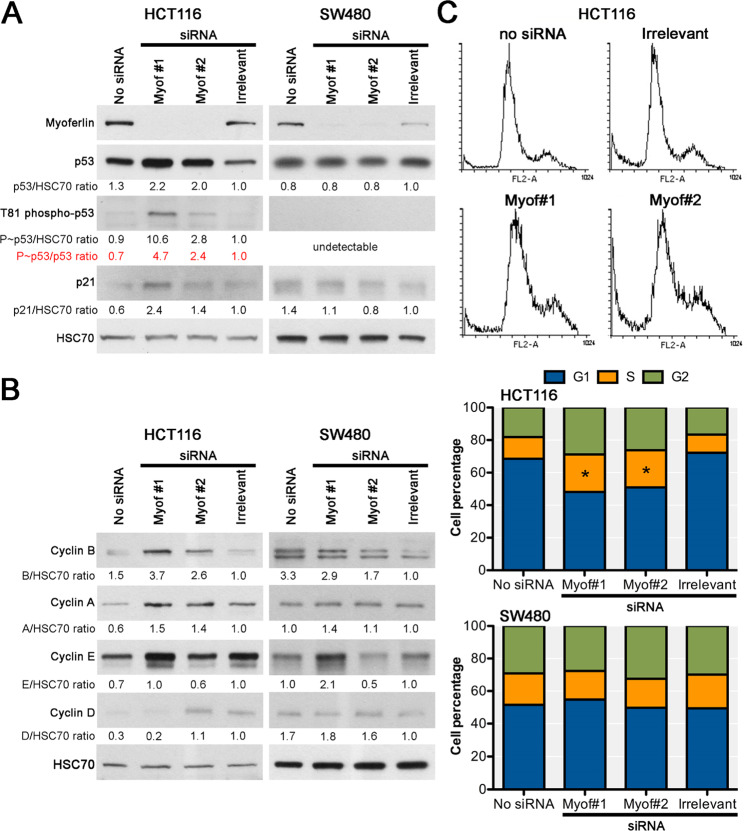
Effects of myoferlin silencing on p53 activation and cell cycle progression. **a** p53 activation by Thr81 phosphorylation and subsequent p21 abundance were evaluated in HCT116 and SW480 48h after myoferlin silencing. **b** Cyclin abundance was evaluated by western-blot in HCT116 and SW480 48h after myoferlin silencing. Total protein extracts (10 μg) were subjected to SDS-PAGE followed by western blot analysis with specific antibodies. HSC-70 was used as a loading control. **c** Cell cycle was analyzed by flow cytometry after propidium iodide incorporation in HCT116 and SW480 48h after myoferlin silencing. Distribution of FL2 fluorescence (propidium iodide) was shown in HCT116. Proportion of cells in G1, S or G2 was shown in HCT116 and SW480. One representative experiment out of three is illustrated. *P < 0.05.

**Fig. 5:**
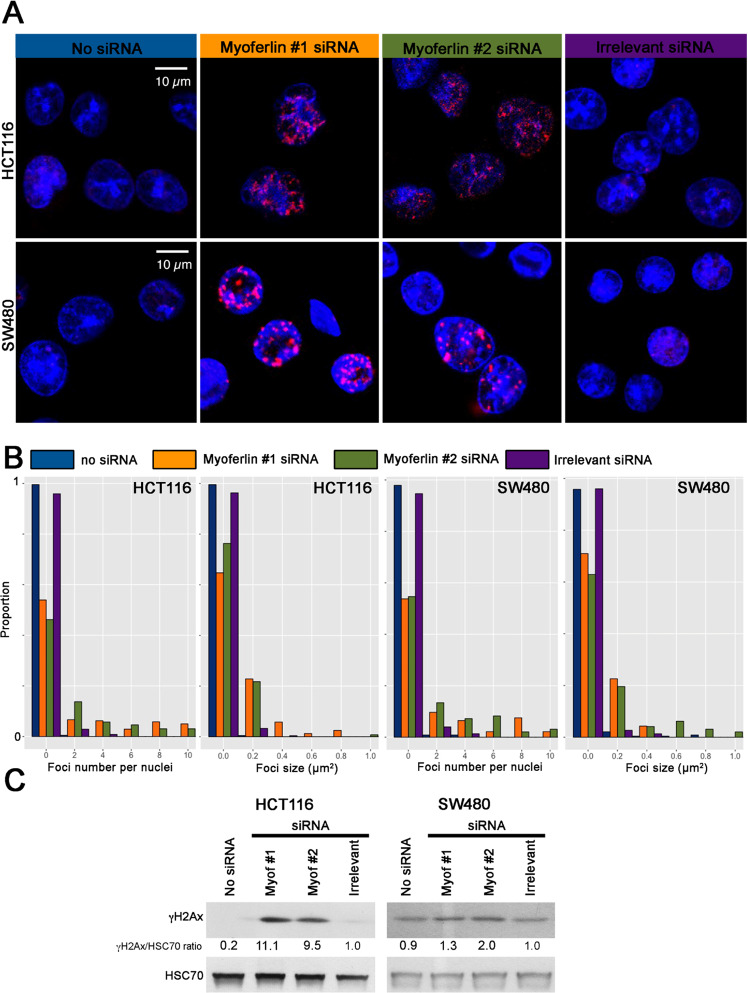
Myoferlin-silencing induces a DNA damage response. **a** HCT116 and SW480 cell lines, silenced for myoferlin during 48h, were stained for γH2Ax and observed under a confocal microscope. **b** γH2Ax foci number and size were quantified using ImageJ. Number and size distributions were established (*n* > 210 nuclei). **c** γH2Ax abundance was evaluated by western-blot in HCT116 and SW480 48h after myoferlin silencing. Total protein extracts (10 μg) were subjected to SDS-PAGE followed by western blot analysis with specific antibodies. HSC-70 was used as a loading control.

