# Supplementary material for: Human colon cancer cells highly express myoferlin to maintain a fit mitochondrial network and escape p53-driven apoptosis
Source: Oncogenesis. 2019 Mar 8;8(3):21. doi: 10.1038/s41389-019-0130-6 (PMC6408501; doi:10.1038/s41389-019-0130-6)
Supplement: Supplementary file 1 — Supplemental legends [file 41389_2019_130_MOESM1_ESM.pdf]

Supplemental Fig. 1 High myoferlin expression is not associated with TNM categories.

Supplemental Fig. 2 Myoferlin expression in COAD cell lines and impact of its silencing on oxygen consumption rate (OCR) and mitochondrial network. **a** Clustering of colon cancer cell lines according to myoferlin expression. **b** Maximal OCR, **c** baseline and stressed (oligomycin + FCCP) OCR, **d** OCR metabolic potential as a percentage of baseline condition for HCT116 cell line. Each bar represents mean ± SD, n = 3. **e** Mean number of networked mitochondria, **f** mean network length, **g** ratio between the number of networked and individual mitochondria in HCT116 cell line. **h** Maximal OCR, **i** baseline and stressed (oligomycin + FCCP) OCR, **j** OCR metabolic potential as a percentage of baseline condition for SW480 cell line. Each bar represents mean ± SD, n = 3. **k** Mean number of networked mitochondria, **l** mean network length, **m** ratio between the number of networked and individual mitochondria in SW480 cell line. ***P < 0.001, **P < 0.01, *P < 0.05.

Supplemental Fig. 3 Myoferlin silencing induced mitochondrial ROS accumulation in HCT116 and SW480 cell lines. HCT116 and SW480 cell lines transfected for 48h with myoferlin siRNA were stained with Mitosox probe and observed under a confocal microscope with reflection (red) and transmission detection (bright).

Supplemental Fig. 4 Myoferlin silencing reduced HCT116 growth on chorioallantoic membrane. **a** Macroscopic top and side view of representative tumours. **b** Tumour size calculated as an ellipsoid volume. n > 6 in each group, ****P < 0.0001, **P < 0.01.

Supplemental Fig. 5 Characterisation of HCT116 Δp53 and prima-1 treated SW480 cells. Total protein extracts (10 μg) were subjected to SDS-PAGE followed by western blot analysis with specific antibodies. HSC-70 was used as a loading control.

Supplemental Fig. 6 Myoferlin-expression is associated to p53 mutation status in colon cancer patients (TCGA-COAD). Myoferlin gene expression was analysed according to the p53 mutation status.
