# Supplementary figures and images for: Human colon cancer cells highly express myoferlin to maintain a fit mitochondrial network and escape p53-driven apoptosis
Source: Oncogenesis. 2019 Mar 8;8(3):21. doi: 10.1038/s41389-019-0130-6 (PMC6408501; doi:10.1038/s41389-019-0130-6)

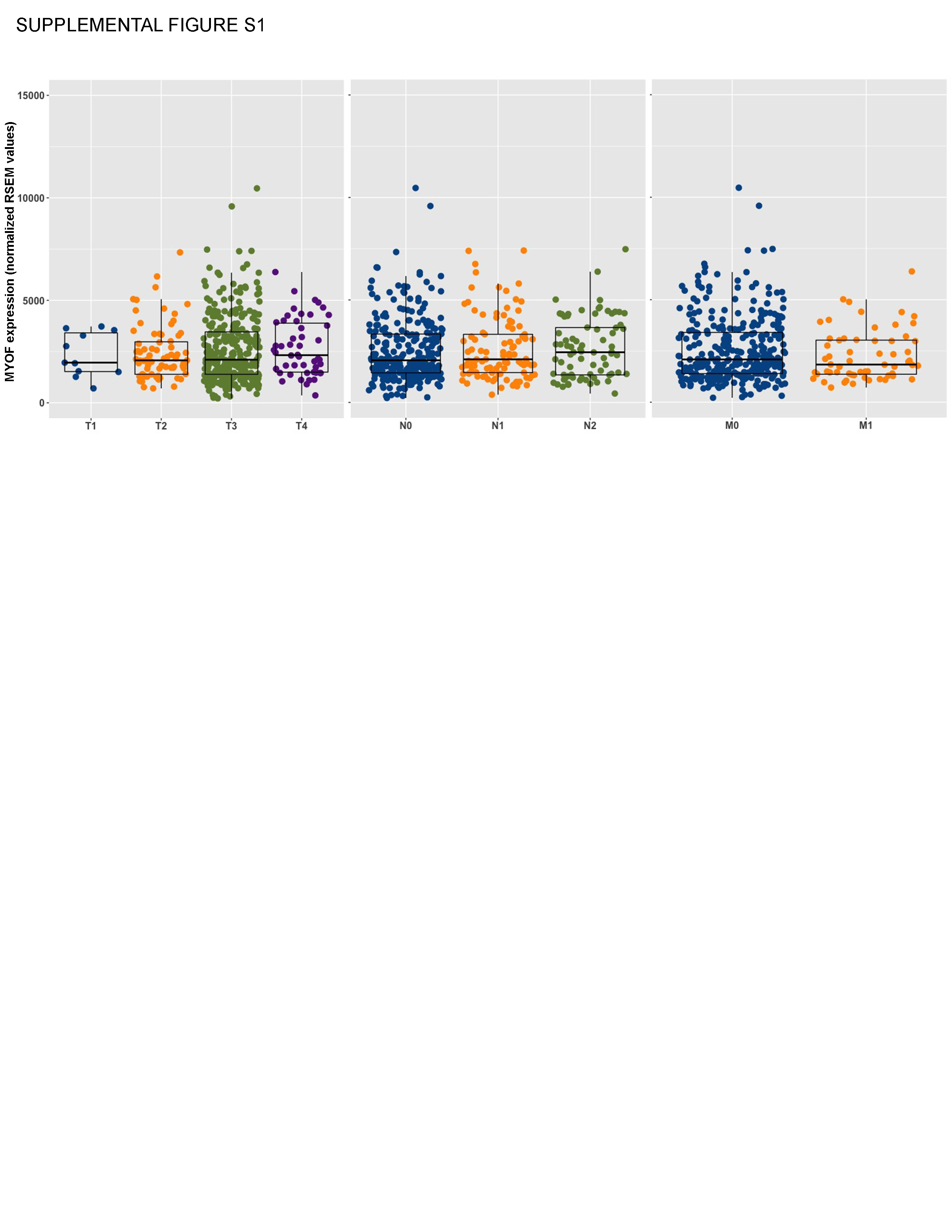

Supplement: Supplementary file 2 — Supplemental Figure 1 [file 41389_2019_130_MOESM2_ESM.tif]

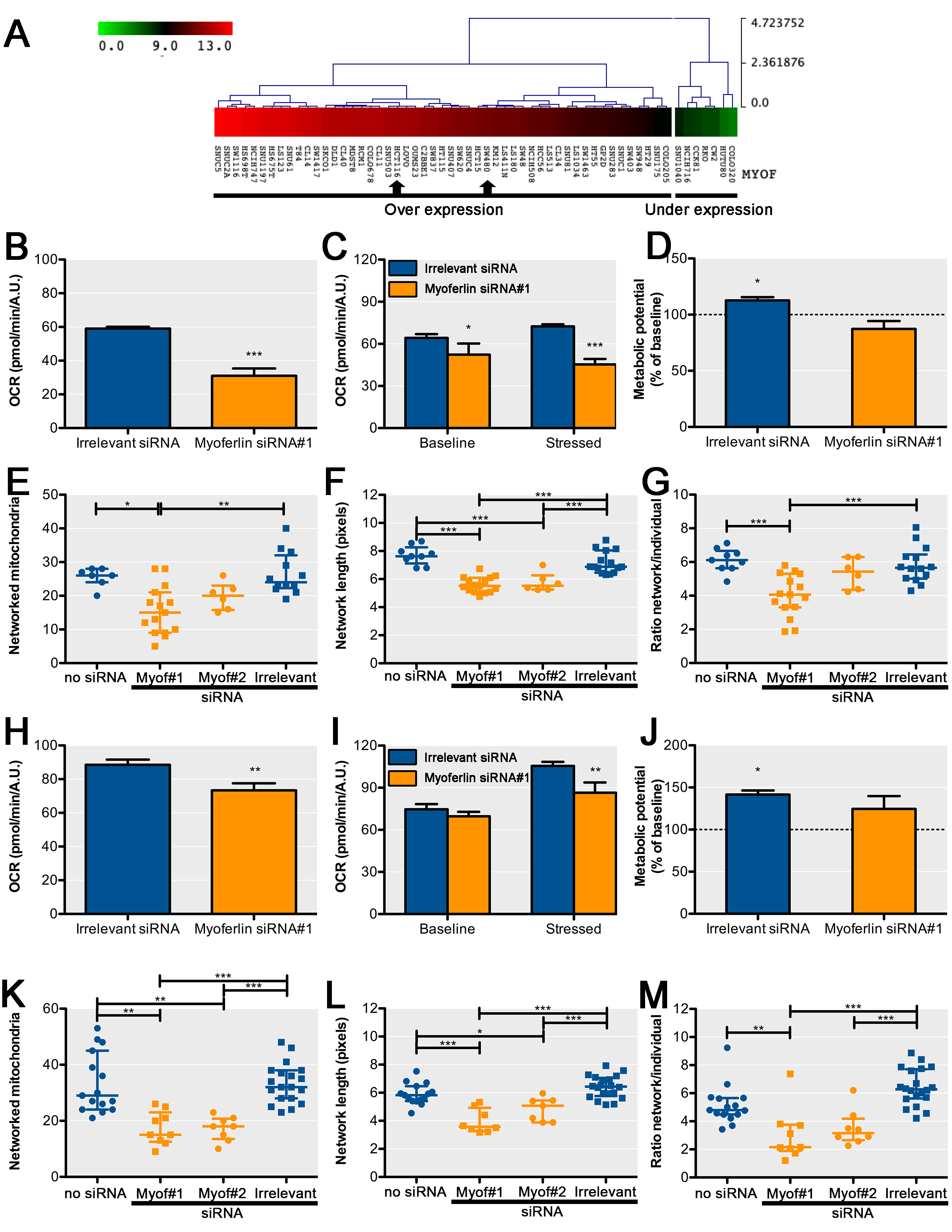

Supplement: Supplementary file 3 — Supplemental Figure 2 [file 41389_2019_130_MOESM3_ESM.tif]

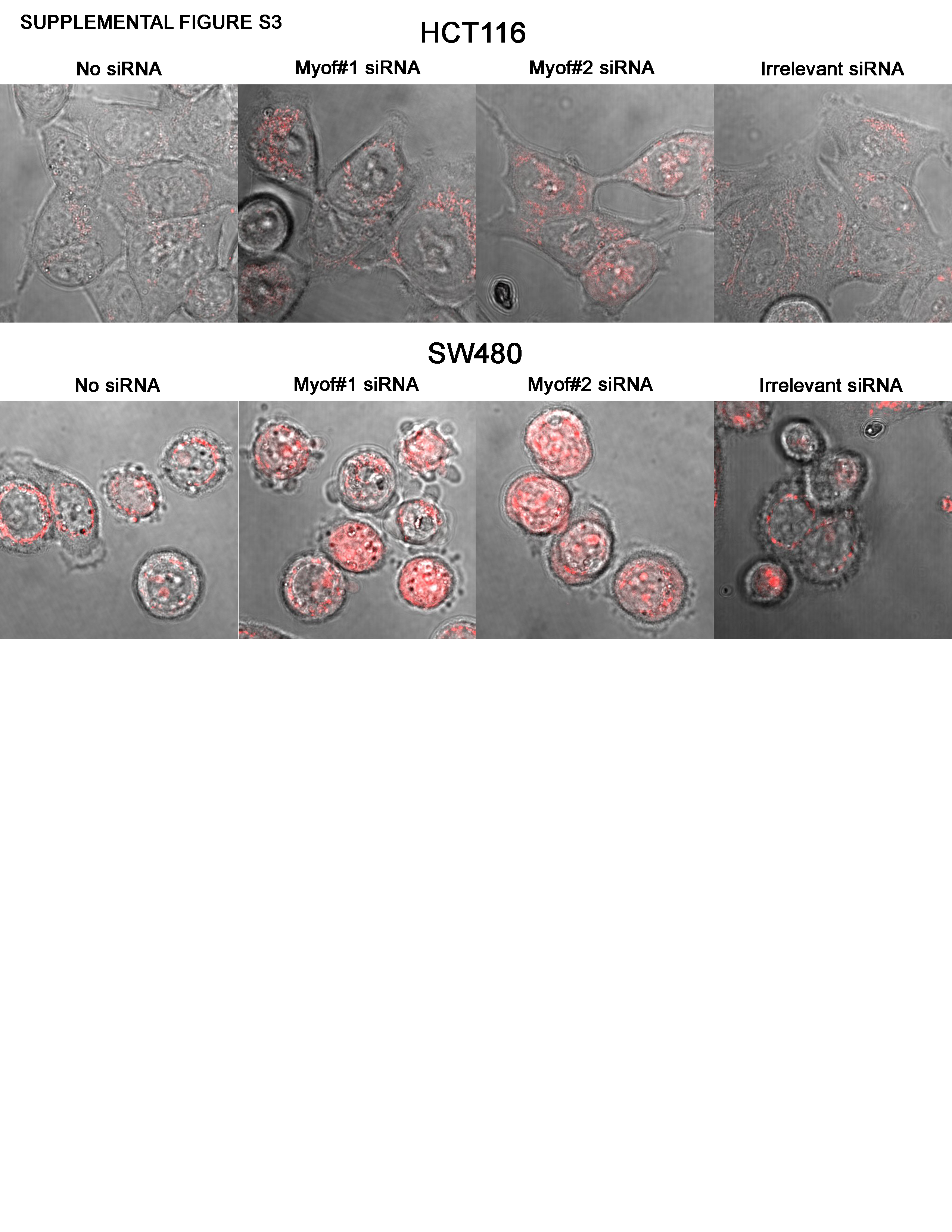

Supplement: Supplementary file 4 — Supplemental Figure 3 [file 41389_2019_130_MOESM4_ESM.tif]

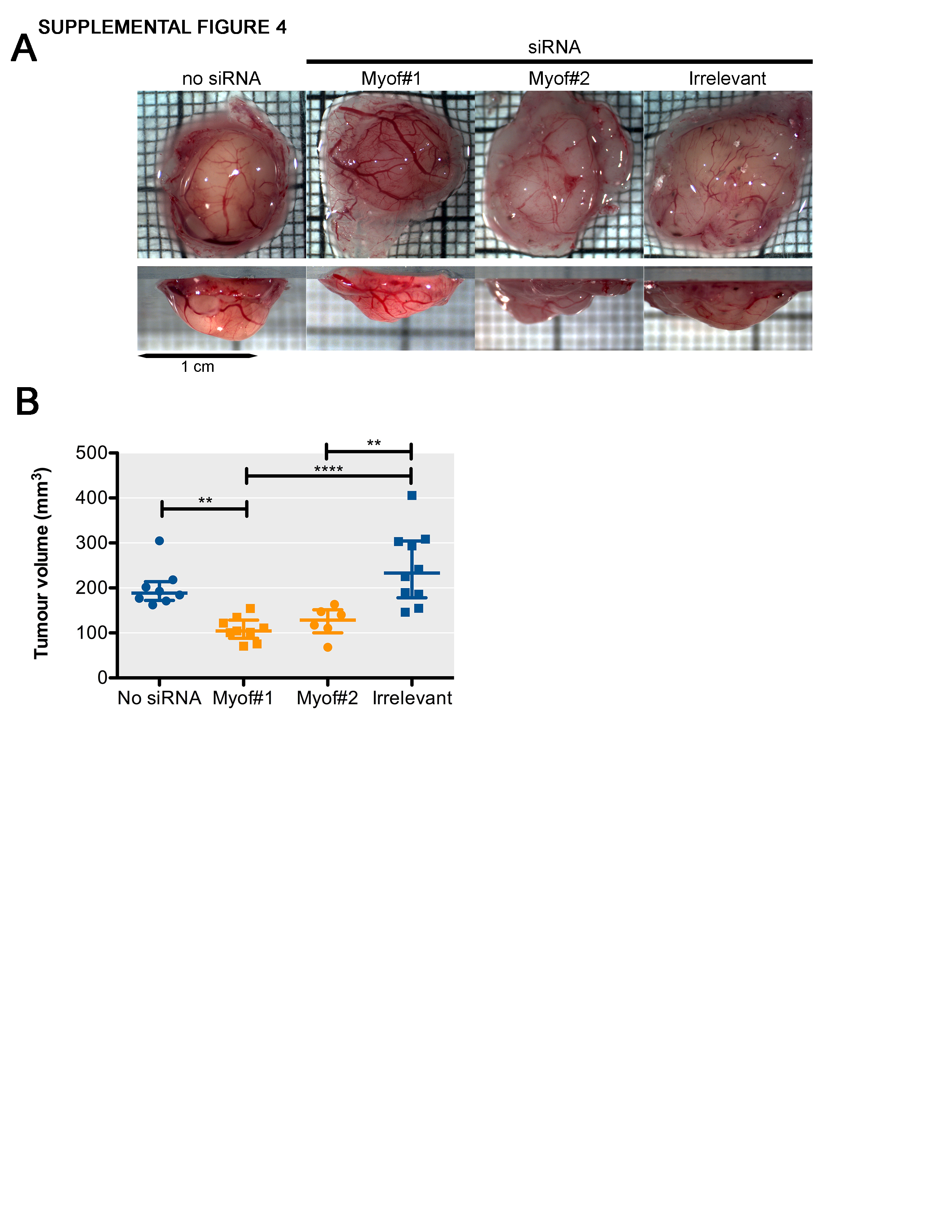

Supplement: Supplementary file 5 — Supplemental Figure 4 [file 41389_2019_130_MOESM5_ESM.tif]

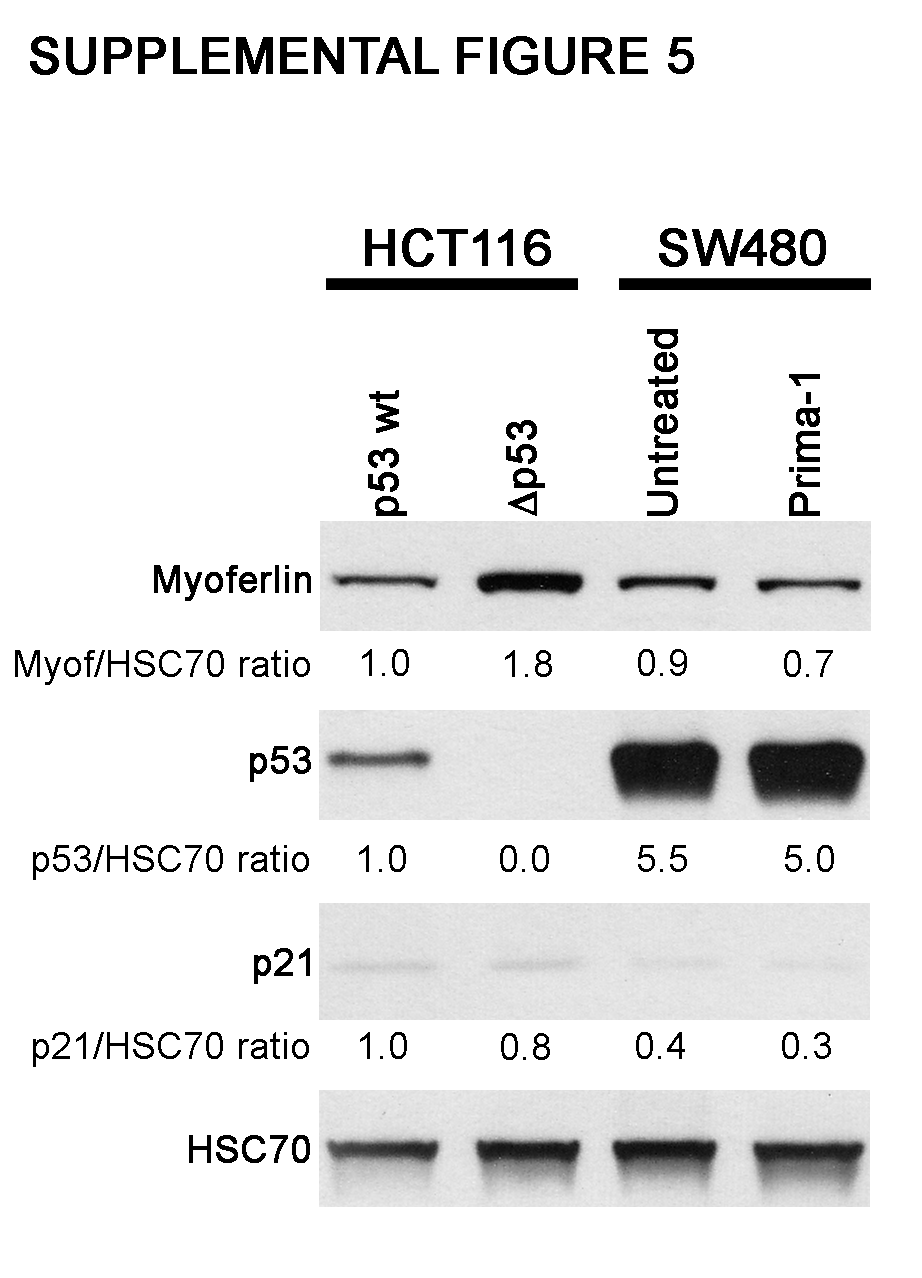

Supplement: Supplementary file 6 — Supplemental Figure 5 [file 41389_2019_130_MOESM6_ESM.tif]

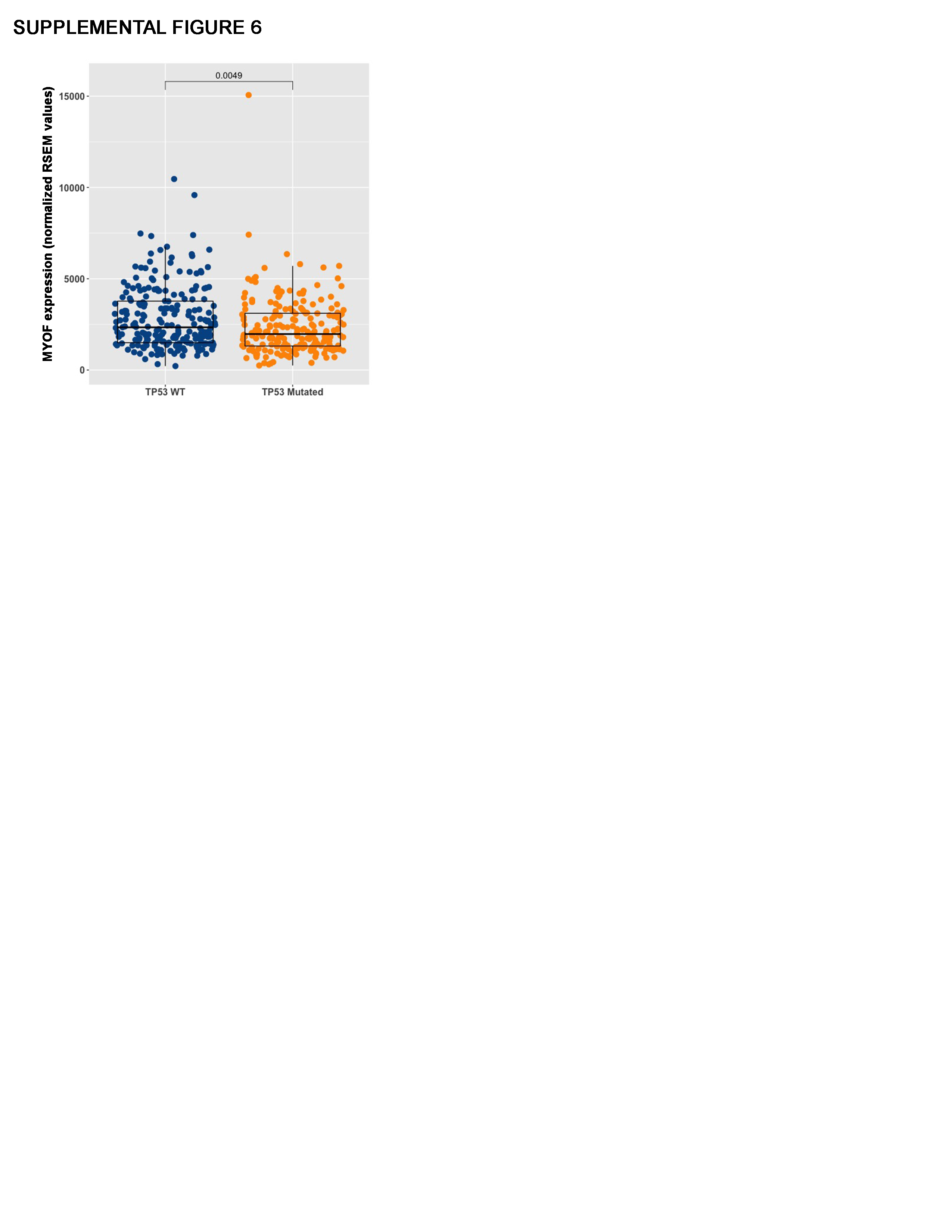

Supplement: Supplementary file 7 — Supplemental Figure 6 [file 41389_2019_130_MOESM7_ESM.tif]
